# Your Fear is My Fear: The Relationship Between Parental and Offspring Anxieties

**DOI:** 10.1007/s10578-020-01060-y

**Published:** 2020-09-18

**Authors:** Dirk Adolph, Jürgen Margraf, Silvia Schneider

**Affiliations:** Mental Health Research and Treatment Center, Ruhr-University Bochum, Massenbergstrasse 9-13, 44847 Bochum, Germany

**Keywords:** Anxiety disorders, Familial transmission, Everyday-life anxiety, Population sample, Epidemiology

## Abstract

Contrary to the well-documented link between parental and offspring clinical anxiety, little is known about the relationship between parental everyday-life anxieties (e.g., concerning family, finances, health) and offspring anxieties. To close this gap, we assessed the frequency of parental symptoms of DSM-IV anxiety disorders and everyday-life anxieties, as well as the frequency of offspring anxiety symptoms in a representative sample by self-report. Parents reported that 48.4% of the children were free of specific symptoms of DSM-IV anxiety disorders within the last 12 months, 39.2% showed low symptom load (1–3 symptoms) and 12.4% were moderately or severely strained (4–10 symptoms). Replicating previous studies, parental DSM-IV symptoms increased offspring risk for the same symptoms. In addition, parental everyday-life anxieties showed a positive relationship with offspring symptom severity. Demographic variables (female sex, low socioeconomic status and younger age) and parental anxiety markers explained 18% of variance in offspring symptom severity. The data are discussed in light of current models of familial transmission.

## Introduction

Anxiety disorders (AD) are among the earliest mental disorders occurring across the lifespan, showing a median age-of-onset of 11 years [1]. Anxiety disorders are highly prevalent and the most common mental disorder in children and youths (6.5%) worldwide [2]. If not treated, childhood anxiety disorders can lead to other mental disorders, for example other anxiety disorders, substance abuse or depression [3, 4]. For example, a recent longitudinal study by Lieb et al. following 3021 youths in Germany found that adolescents diagnosed with specific phobia have an increased risk of developing panic disorder, generalized anxiety disorder, post-traumatic stress disorder, obsessive compulsive disorder as well as major depression, eating disorders and chronic pain [5]. Moreover, anxiety disorders are highly persistent. In the same study, 41% of adolescents with specific phobia and 35% with social phobia at age 14 were diagnosed with the same disorder 10 years later [6, 7]. To understand the development of an anxiety disorder, it is essential to take into account the various influencing factors. Within the current study, we aimed to address the role of different forms of parental anxiety in the occurrence of anxiety symptoms in children.

Especially familial relationships and environment can greatly influence the development of an anxiety disorder. Such transgenerational transmission of anxiety is explained by genetic factors as well as family factors related to characteristics of parent–child interactions (for a comprehensive overview see [8]). For example, having a first-degree family member with an anxiety disorder is among the most consistent and well-replicated risk factors for anxiety disorders [9]. Numerous studies have shown a strong association between parental and offspring anxiety disorders [10, 11] and most studies confirm that parental anxiety disorders are among the most powerful predictors of anxiety in children [12]. For example, children of parents with anxiety disorders show a higher risk of developing an anxiety disorder than children without anxious parents [12] and children with an anxiety disorder are two to three times more likely to have at least one parent with a current or lifetime anxiety disorder than control children [13]. Moreover, data suggest that at least for a subset of anxiety disorders (i.e., panic, social anxiety disorder, and generalized anxiety disorder) children’s risk is specifically enhanced when parents suffer from the same disorder [13, 14]. Taken together, the association between parental clinical anxiety and child anxiety is well established [15] and particularly pronounced in comparison to familial transmission of other disorders (for a recent review on putative mechanisms of familial transmission see [16]).

In contrast, little is known about the relationship between child anxieties and parental everyday-life anxieties (those anxieties focusing on aspects of everyday-life, concerning for example financial affairs, health, family, political and environmental situation). Indeed, everyday-life anxieties are rather common. For example, there are indications that everyday-life anxieties in North America have increased by approximately one standard deviation over the last half of the century [17]. Approximately one third of adults within the general population suffer from everyday-life anxieties, and approximately 5–7% are severely strained [18]. It has been argued that parents’ anxious behavior and expressed anxiety promote and maintain child anxiety through modeling and instruction learning, that is, catastrophizing and over-attribution of threat [19]. Thus, it is likely that also everyday-life anxieties, along with clinically relevant anxieties, increase the risk of developing childhood anxieties. However, to date, this question has been largely neglected.

### Aim of the Present Study

With the present study, we aimed at closing this gap and assessed the predictive power of different types of parental anxieties, including everyday-life anxieties, on the occurrence of offspring anxieties. We recruited a large representative sample from the general population. We assessed (1) the number of symptoms of DSM-IV anxiety disorders parents had experienced during the last 12 months and during their life time (e.g., fear of specific objects or situations, panic symptoms), (2) the amount of disorder-unspecific (bodily) anxiety symptoms (dizziness, wobbliness in knees, faintness, etc.), as well as (3) the amount of anxieties concerning everyday-life contents (i.e., everyday-life anxieties: fears about the families’ well-being, financial situation, fear of war or terror, etc.). In the same vein, we assessed the number of symptoms of DSM-IV anxiety disorders shown in the children of these parents during the last 12 months.

Within the scope of the present manuscript, we first report the frequency of symptoms of DSM-IV anxieties in children. In a second step, we calculate the predictive value of parental DSM-IV anxiety symptoms and bodily symptoms for the occurrence of offspring DSM-IV symptoms. Based on the literature, we hypothesize overall high concordance in symptom occurrence between parental and offspring DSM-IV symptoms [16]. That is, the occurrence of a specific parental symptom of DSM-IV anxiety disorders increases the risk for the occurrence of the same symptom in these parents’ offspring. Finally, we test whether the amount of parents’ everyday-life anxieties is predictive of the occurrence of anxiety symptoms in children. Based on the theoretical assumptions presented above, we hypothesize that parental everyday-life anxieties would also (i.e., in addition to clinically relevant anxieties) be associated with offspring anxieties.

We used a new time- and cost-efficient screening instrument to assess the occurrence of symptoms of DSM-IV anxiety disorders and the amount of everyday-life anxieties, as well as the amount of somatic anxiety symptoms in the general population (for a comprehensive description see [18]). We have previously shown that the results of this instrument in a representative sample correspond with the findings of epidemiological studies on the prevalence of anxiety disorders [18].

## Methods

### Participants and Participant Assessment

In order to assess the association between parental and child anxieties in the general population, we recruited a sample representative to the German population (i.e., in terms of residency within Germany’s federal states, household size, age groups, and gender distribution). Data collection was completed between March 26th 2013 and September 22nd 2014 and carried out by an institute for market and social research (USUMA GmbH, Berlin). Participants were recruited in two subsamples using a mixed methods approach (sampling system for telephone surveys, “Easy Sample”, Arbeitsgemeinschaft Deutscher Marktforschungsinstitute e.V., ADM, Germany). Response rates were 48% and 57%, respectively. A total of n = 1008 individuals was interviewed by telephone, while a total of n = 2308 individuals completed the survey either online or using a paper and pencil version. Thus, our initial adult sample consisted of n = 3316 German-speaking residents within the Federal Republic of Germany. The age of respondents ranged between 18 and 98 years (M = 53, SD = 18), n = 1579 (47.6%) were male, and n = 1737 (52.4%) were female. All respondents were initially asked to answer questions concerning anxieties during the course of their life. After finishing the adult survey,^1^ those respondents who reported having children, were additionally asked to answer questions focusing on the anxieties of their offspring. Although we are aware of the benefits of a multi-informant approach, we refrained from interviewing the children directly. This was done mainly because we sought a large, representative sample, and a multi-informant approach would have largely increased the expenses of the current study, which in turn would have led to significantly smaller sample sizes.

Out of the 3316 adult respondents, n = 560 (16.9% of all participants) reported having at least one child between 5 and 18 years of age, summing up to a total of n = 924 children-parent pairs within the current sample (see Table 1 for information about the number of children per family). Respondents with children were between 21 and 83 years of age (M = 45, SD = 8), n = 380 (41.1%) were male and n = 544 were female (58.9%). The respondents’ children had a mean age of M = 11.5 years (SD = 3.7), and n = 451 (48.8%) were male, while n = 466 (50.4%) were female. Seven parents refused to report their child’s age. The study was conducted in agreement with the Declaration of Helsinki and approved by the Ethics Committee of the Faculty of Psychology at Ruhr University Bochum. Informed consent was obtained from all participants.

**Table 1:** Frequencies of family size within the current sample

Number of children per family	Frequency of these families within the final sample	Sum of children (%)
7	1	7 (0.8%)
6	0	0 (0%)
5	2	10 (1.1%)
4	13	52 (5.6%)
3	42	126 (13.6%)
2	228	456 (49.3%)
1	274	274 (29.6%)
	∑	925

### Assessment of Parental Anxieties

#### Disorder-Unspecific (Bodily) Anxiety Symptoms

To assess disorder-unspecific bodily symptoms of anxiety, the Short Questionnaire for Anxiety Disorders [20] was used. It represents a short form of the Beck Anxiety Inventory German version [21] and measures the intensity of six mostly bodily symptoms of anxiety and was successfully used in previous studies to estimate prevalence rates [22] and bodily anxiety symptoms in the general population [18]. The questionnaire contains six items assessing the suffering from several mainly physiological anxiety symptoms within the last 7 days (wobbliness in knees or legs, dizzy or lightheaded, shaky or unsteady, hands trembling, scared, feeling of faintness) on a 4-point scale (0 = not at all, 1 = mildly, 2 = moderately, it was very uncomfortable but I could bear it, 3 = severely, I could hardly bear it). Joint occurrence of these symptoms indicates the likelihood of an anxiety disorder. The sum score of the scale can vary between 0 and 18 points, where values between 0 and 3 are interpreted as “no disorder”, values between 4 and 6 as “potential disorder” and values greater than 6 as “definite disorder”. Internal consistency of the scale in the present study was *CRα* = 0.85.

#### DSM-IV Disorder-Specific Symptoms of Parental Anxiety

The occurrence of disorder-specific anxiety symptoms according to DSM-IV-TR was assessed with seven questions closely following the basic questions (A-criterion) of a German standardized clinical interview for mental disorders (Diagnostisches Interview bei Psychischen Störungen, DIPS) [23] representing the core symptoms of various anxiety disorders. Assessing core symptoms represents a time-efficient screening method with appropriate diagnostic accuracy [24]. In the present study, the respondents were asked whether the relevant symptoms occurred within the last 12 months, whether the symptoms had occurred longer than 12 months, or whether they had never occurred.

#### Anxieties Regarding Different Aspects of Everyday-life

To assess the intensity of anxieties related to aspects of everyday-life, participants responded on a scale from 0 to 3, analogously to the Short Questionnaire for Anxiety Disorders, whether they currently feel anxious or worry about: (1) occupational affairs like school, studies, work, (2) familial affairs, (3) friends, (4) neighbours/ neighbourhood, (5) financial affairs, (6) own and family health, (7) Internet, social networks, (8) personal future or the future of their children, (9) war, terrorism, (10) environmental disasters, (11) general economic situation in Germany and Europe, and (12) general political situation in Germany and Europe. The 12 items can be combined into three subscales (range 0–3) assessing “political and economic environment” (Items 9, 10, 11, 12, *CRα* = 0.89), “own person and family” (Items 1, 2, 5, 6, 8, *CRα* = 0.76) and “extended personal environment” (Items 3, 4, 7, *CRα* = 0.39) [15]. The subscale “extended personal environment” yielded insufficient internal reliability in the current sample and was therefore excluded from further analyses.

### Assessment of Demographic Variables and Socioeconomic Status

Apart from age, sex, present marital status and household size (number of persons permanently living in the household), current occupation (11-level categorical selection), educational level (6-level categorical selection), and net household income were recorded in order to determine socioeconomic status. Socioeconomic status (SES) was computed by means of the variables occupation, net household income and level of education [25, 26]. The current survey considered only the persons who gave answers to all three variables. The resulting sum score can be categorized into “medium socioeconomic status”, “low” and the highest quintile “high socioeconomic status” [25, 26].

### Assessment of Childhood Anxieties

Because of feasibility reasons (see above), the occurrence of specific anxiety symptoms according to DSM-IV-TR was assessed via parental report with questions closely following the basic questions (A-criterion) of Kinder-DIPS [27] representing the core symptoms of the respective anxiety disorders. In sum, the present study assessed symptoms of offspring separation anxiety, specific phobia, social phobia, generalized anxiety, panic, agoraphobia, trauma, obsessive compulsive disorder, as well as school anxiety/ school absenteeism and fear of strangers. Offspring clinical anxieties were assessed with similar scales as adult clinical anxieties. In addition, for a subset of children (n = 268 children^2^), we assessed whether parents had observed changes in the child’s behaviour (any behavioural impairment associated with the situation, for example avoidance behaviour, discomfort, etc.) after initially recognizing the presence of the respective symptom.

## Data Reduction and Analysis

The first aim of the study was to assess the frequency of childhood anxiety symptoms. Therefore, we calculated the cumulated frequency of occurrence over last 12 months as well as during the entire lifetime separately for each DSM-IV disorder specific symptom.

The second aim of the study was to test whether parental DSM-IV symptoms and bodily symptoms predict offspring DSM-IV anxiety symptoms (see ‘Results’ section: Predicting childhood anxieties with Parental DSM-IV symptoms and bodily anxiety symptoms). Therefore, odds ratios for the 12-month occurrence of each childhood clinical anxiety symptom (i.e., panic, separation anxiety, etc.) as dependent variable were assessed by calculating logistic regressions including both the respective specific adult DSM-IV anxiety symptom and the amount of parental bodily symptoms as predictors (for example in the case of offspring panic symptoms, parental panic symptoms as well as parental unspecific anxiety symptoms were included as predictors).

Next, we determined the association between parental everyday-life anxieties and childhood anxieties (see ‘Results’ section: Predicting childhood anxieties with Parental everyday-life anxieties). Therefore, we first calculated the total offspring symptom load by summing the number of specific anxiety symptoms for each child during the last year [18]. Then, we calculated regression analyses using the total offspring symptom load score as dependent variable, and the two subscales of the parental everyday-life anxieties questionnaire as predictors. In case of significant regression equations, we also calculated single regression analyses including the individual items of the everyday-life subscale as predictors and total offspring symptom load as dependent variable.

Moreover, we also controlled the regression for the frequency of occurrence of parental GAD symptomatology and severity of parental bodily symptoms of anxiety. We controlled for these factors to differentiate everyday-life anxieties from worrying common within generalized anxiety disorder (GAD). GAD is associated with repetitive worry about everyday-life issues and the content of worries do not substantially differ between people suffering from GAD and healthy controls [28]. However, previous research has shown that accompanying physical anxiety symptoms and uncontrollability of worries play a pivotal role in differentiating generalized anxiety disorder from non-pathological worrying [28]. If parental everyday-life anxieties indeed represent an independent concept, regression coefficients of parental everyday life anxieties should remain significant after including these variables in the regression equation.

Finally, hierarchical linear regressions were performed to assess unique and shared variance of DSM-IV symptoms and everyday-life anxieties (see ‘Results’ section: Predicting childhood anxieties with parental clinical and everyday-life anxieties). In the first step, demographic variables were included as control variables. Total parental symptom load (analogously to total offspring symptom load this was calculated as the sum of all reported DSM-IV symptoms for each parent) and parental everyday-life anxieties were added to the model in the second step. This regression model enabled us to assess the unique variance explained by each of the predictors (i.e., demographic variables, parental anxieties). All analyses were performed using SPSS 21.0.

## Results

### Frequency of Childhood Anxieties

Table 2 gives an overview of the individual frequencies of common clinically relevant anxieties in children reported by the parent. The 12-month rate of occurrence ranged from 2.1 to 9% for most anxiety symptoms to up to 15–30% for symptoms of panic, generalized anxiety and specific phobias (see Table 2). When additionally taking behavioral avoidance into account, rates were considerably lower, and ranged from approximately 0.6–5.1% for trauma, OCD, Panic/Agoraphobia, social phobia and separation anxiety, up to 7.5–11% for generalized anxiety and specific phobias (see Table 2). Considering the individual symptom load, children were not affected equally. While in sum 47.4% of all children were free of any symptoms within the last 12 months, 41.7% showed low symptom load (1–3 specific anxiety symptoms), 8.6% showed moderate symptom load (4–6 specific anxiety symptoms within the last 12 months), and a total of 1.5% were severely strained (7–10 specific anxiety symptoms within the last 12 months). Girls (*M* = 1.46; *SD* = 1.75) showed a significantly higher total symptom load as compared to boys (*M* = 1.08; *SD* = 1.59), *F*_(1/742)_ = 10.38, *p* = 0.001, *f* = 0.12*.* Also, higher symptom load was associated with lower socioeconomic status, *F*_(1, 742)_ = 5.44, *p* = 0.020, *f* = 0.08, and a childs younger age, *F*_(1,742)_ = 27.77, *p* < 0.001, *f* = 0.19.

**Table 2:** Symptom frequency of offspring anxieties

Symptom	Frequency 12 month without impairment (%)	Frequency 12 month with impairment (%)	Frequency lifetime (%)	Frequency lifetime with impairment (%)
Separation anxiety	16.1	7.1	43.2	10.1
Specific phobia	29.2	15.3	54.9	17.5
Social phobia	9.0	4.1	19.8	6.0
Generalized anxiety	24.5	10.4	39.6	15.7
Panic	14.8	10.4	25.3	12.3
Agoraphobia	7.6	6.0	15.7	7.5
Trauma	2.1	1.9	1.2	7.1
Obsessive/compulsive behavior	6.9	3.0	12.9	4.9
School absentism	5.2	2.2	8.9	2.6
Anxiety of strangers	7.0	3.7	21.5	4.9

### Predicting Childhood Anxieties with Parental DSM-IV Symptoms and Bodily Anxiety Symptoms

For most DSM-IV symptoms, the 12-month occurrence in parents was associated with an increased risk of their offspring to experience the same symptoms (see Table 3 for an overview). Predictive power was especially pronounced for symptoms of trauma, agoraphobia, and obsessive-compulsive disorder. Parental symptoms of panic, agoraphobia, specific phobia, social anxiety, generalized anxiety, obsessive compulsive disorder, as well as trauma were associated with a moderate to high risk of observing these disorder-specific symptoms also in their children. Finally, parental panic symptoms significantly enhanced the risk of offspring separation anxiety, χ^2^_(1)_ = 10.48, p = 0.001, R^2^ = 0.02, OR = 1.87; CI [1.29, 2.71].

**Table 3:** Prediction of childhood anxieties with unspecific and specific parental anxiety symptoms occurring during the past 12 months

Dependent variable	Bodily symptoms				DSM-IV symptoms			
	χ^2^_df1_	R^2^	B	OR_[CI]_	χ^2^_df1_	R^2^	B	OR_[CI]_
Panic symptoms	28.91***	0.06	0.15***	1.16_[1.10/1.22]_	20.40***	0.04	0.89***	2.43_[1.67/3.55]_
Agoraphobic symptoms	16.89***	0.04	0.14***	1.15_[1.08/1.23]_	6.13*	0.02	0.92**	2.50_[1.28/4.91]_
Symptoms of specific phobia	38.39***	0.06	0.15***	1.16_[1.11/1.22]_	24.96**	0.04	0.79***	2.20_[1.62/2.99]_
Social anxiety symptoms	22.90***	0.06	0.15***	1.17_[1.10/1.24]_	8.24**	0.02	0.67**	1.96_[1.24/3.10]_
Symptoms of generalized anxiety	13.84***	0.02	0.09***	1.10_[1.05/1.15]_	19.25***	0.03	0.76***	2.13_[1.53/2.97]_
Trauma	0.66^n.s.^	0.00	0.06^n.s.^	1.06_[0.93/1.21]_	5.56*	0.03	1.88***	6.53_[1.78/23.92]_
Obsessive compulsive thoughts and actions	0.17^n.s.^	0.00	0.02^n.s.^	1.02_[0.94/1.11]_	6.57*	0.02	0.78***	2.18_[1.24/3.84]_
Separation anxiety	15.36***	0.03	0.11***	1.12_[1.06/ 1.18]_				
School abstentism	1.09^n.s.^	0.01	− 0.06^n.s.^	0.94_[0.83/1.06]_				
Anxiety of strangers	0.51^n.s.^	0.01	0.06^n.s.^	1.06_[0.91/1.24]_				

*n.s. *non-significant

*p < 0.05; **p < 0.01; ***p < 0.001; for separation anxiety, school absentism and anxiety of strangers data for parents not available

Parental bodily anxiety symptoms were moderately associated with offspring symptoms of panic, agoraphobia, specific phobia, generalized anxiety, as well as separation anxiety (for all analysis: 1.10 < OR < 1.16). There was no association between parental bodily anxiety symptoms and offspring symptoms of trauma, obsessive compulsive disorder, school absenteeism and fear of strangers.

Finally, the sum of parental anxiety symptoms (total parental symptom load) significantly predicted total offspring anxiety symptom load, F_(1,903)_ = 87.15, p < 0.001, beta = 0.300, p < 0.001, R^2^ = 0.09. This association was independent of the child’s age, sex or family’s socioeconomic status, F_(4,732)_ = 30.30, p < 0.001, beta = 0.312, p < 0.001, R^2^ = 0.09.

### Predicting Childhood Anxieties with Parental Everyday-Life Anxieties

Regression analysis predicting total offspring symptom load with the subscales “anxieties concerning their own person and family”, as well as “anxieties concerning the political and economic environment” revealed a significant regression equation. Total offspring symptom load, F_(2,915)_ = 46.51, p < 0.001, R^2^ = 0.09, was significantly predicted by parental everyday-life anxieties concerning their own person and family, β = 0.302, p < 0.001) but not by anxieties concerning the political and economic environment, β = 0.005, p = 0.893. This effect was independent of the presence of parental GAD symptomatology and the severity of parental bodily symptoms of anxiety, F(3,899) = 38.60, p < 0.001, R^2^ = 0.114. 1, β = 0.236, p < 0.001.

To assess which items from the subscale “own person and family” were responsible for the overall predictions of total offspring symptom load, regressions including the single items of the subscale were performed. These analyses revealed that all items of the scale were significant predictors for offspring total symptom load, including anxieties concerning occupational affairs, F_(1,913)_ = 25.40, p < 0.001, R^2^ = 0.03, β = 0.165, familial affairs, F_(1,915)_ = 58.08, p < 0.001, R^2^ = 0.06, β = 0.244, financial affairs, F_(1,915)_ = 27.54, p < 0.001, R^2^ = 0.03, β = 0.171, own and family health, F_(1,915)_ = 50.78, p < 0.001, R^2^ = 0.05, β = 0.229, personal and children’s future, F_(1,912)_ = 73.75, p < 0.001, R^2^ = 0.08, β = 0.274.

### Predicting Childhood Anxieties with Parental Clinical and Everyday-Life Anxieties

As expected, hierarchical linear regression indicated that all demographic variables significantly predicted offspring total symptom load (see Table 4, regression model Step 1) explaining about 5% of the variance. Entering parental anxieties (see Table 4, regression model Step 2) explained another 13% of the variance in offspring symptom load. However, entering parental anxieties also explained the variance added by socioeconomic status, which was no longer a significant predictor of the offspring total symptom load. In sum, the current model explained about 18% of the variance of the offspring total symptom load (see Table 4, regression model Step 2).

**Table 4:** Prediction of offspring total symptom load with parental anxieties with clinical content, parental everyday life anxieties as well as sociodemographic variables

Predictor	β	Δ*R*^2^	*F*_*df*_
Step 1		0.05	13.73_(3,731)_***
Age_Child_	− 0.188***		
Sex_Child_	0.113**		
SES	− 0.085*		
Step 2		0.13	26.65_(6728)_***
Age_Child_	− 0.167***		
Sex_Child_	0.087		
SES	− 0.033^n.s.^		
EL Fam_Parent_	0.210***		
Unspecific anxiety symptoms_Parent_	0.055^n.s.^		
Total symptom load_Parent_	0.168***		

*n.s.* non-significant

*p < 0.05; **p < 0.01; ***p < 0.001, SES family = socioeconomic status, EL Fam_Parent_ = parental everydaylife anxiety concerning their own person and family

## Discussion

In the present study, we investigated the frequency of DSM-IV anxiety symptoms in children in a large population sample. Specifically, we were interested in their association with parental DSM-IV and everyday-life anxiety symptoms. Confirming previous research, we found that anxiety symptoms are highly prevalent. Moreover, specific parental DSM-IV symptoms predicted the same DSM-IV symptoms in offspring, confirming high disorder specific concordance as reported previously. Furthermore, beyond parental DSM-IV symptoms, this is the first study presenting initial evidence that parental everyday-life anxieties also significantly predict offspring anxiety symptomatology.

### Frequency of Childhood DSM-IV Anxiety Symptoms

The current data confirm previous research showing that anxiety symptoms are highly prevalent and rarely occur in isolation. While about half of the children were free of symptoms, another 40% of the respondents’ children showed at least one anxiety disorder specific symptom within the last 12 months. A total of 10% were more severely strained, showing between 4 and up to 10 symptoms within this period of time. Occurrence rates for individual symptoms (1.9–29%) were considerably higher than epidemiological data for full-blown anxiety disorders, typically obtained from epidemiological studies using clinical interviews [29, 30]. For example, data from a large nationwide survey suggest prevalence rates of around 10% for childhood anxiety disorders in Germany [31]. Our data clearly show that the occurrence rates of anxiety symptoms exceed prevalence rates for full-blown disorders, indicating that at least a subset of the children in the current study showing large symptom load might not fully meet diagnostic criteria. Indeed, when additionally taking behavioural impairment into account (e.g., adjustment of daily routines, avoidance of specific situations, etc.), 12-month and lifetime occurrence rates in the present study were significantly lower, approaching those found in other epidemiological studies [29, 30]. Previous research suggest the assessment of core symptoms to be a time-efficient screening method with good diagnostic accuracy [24]. However, the current results confirm the importance of assessing additional behavioural impairments along with symptom data [29], whereby functional impairment is of key importance when determining burden severity.

In sum, the current data suggest that DSM-IV anxiety disorders’ core symptoms are highly prevalent, but do not necessarily develop into full-blown disorders. In the case of panic attacks, for example, this phenomenon has been reported in previous studies. Typically, the prevalence of panic attacks is higher than the prevalence of DSM-IV panic disorder alone, being frequently comorbid, especially with other anxiety disorders [32]. The current data suggest that also symptoms of other anxiety disorders occurring outside the symptom complex of a full-blown disorder can lead to considerable impairments in the child’s everyday life behaviour. However, in sum, the current prevalence data for anxiety symptomatology seem plausible based on existing literature - an important prerequisite for the main research question.

### Prediction of Offspring DSM-IV Anxiety Symptoms with Parental DSM-IV Anxiety Symptoms

Our main focus was to determine the influencing role of parental anxieties on offspring anxiety. We found robust associations between parental DSM-IV anxiety symptoms and offspring DSM-IV anxiety disorder symptoms. In detail, a specific parental DSM-IV anxiety symptom significantly increases the probability to observe this symptom also in offspring, with odds ratios ranging from 3 for panic symptoms, specific phobia, generalized anxiety, up to 10 for obsessive compulsive thoughts and behaviours, and 18 for agoraphobia. One exception was the robust association between parental symptoms of panic and childhood separation anxiety, which has been reported previously [33].

Taken together, disorder-specific adult symptoms are more robust predictors for the same anxiety symptoms in offspring than bodily anxiety symptoms, with average odds ratios being about twice as high. These data extend previous findings showing that children of anxious parents are primarily at risk of developing anxiety disorders, rather than of developing any other mental disorder [16]. The current data show that such transgenerational concordance might also apply to individual disorders within the anxiety disorders spectrum. These data confirm previous research showing that parental anxieties seem to be a strong predictor for anxieties in children [34, 35].

### The Influence of Demographic Variables on Offspring DSM-IV Anxiety Symptoms

In addition to robust associations with parental symptom severity, we found child’s younger age and family’s lower socioeconomic status to be associated with higher offspring symptom load. The finding that younger children show more severe anxiety symptomatology is in line with previous reports of a decline in general levels of anxiety over the course of childhood [36–38], probably tied to a shift in the age-specific expression of anxiety symptomatology from symptoms of separation anxiety at a younger age to symptoms of social anxiety during adolescence [37]. In addition to age, lower socioeconomic status was associated with child’s higher symptom load. Low socioeconomic status is associated with a number of childhood adversities and has been shown to negatively affect child development [39]. For example, children and adolescents from families with low socioeconomic status are more likely to show mental health problems than their peers from families with a higher socioeconomic status [40], including an enhanced risk to develop anxiety disorders [41]. In sum, these findings underscore the importance of assessing demographic variables for the understanding of the complex mechanisms within the development of childhood anxiety.

### Prediction of Offspring DSM-IV Anxiety Symptoms with Parental Everyday-Life Anxiety Symptoms

Complementing the findings with respect to clinically relevant anxiety symptoms, we found that also a range of parental everyday-life anxieties show a positive relationship to offspring symptom load. This was especially noticeable for parental anxieties concerning one’s own person and family (i.e., financial affairs, health, personal and familial future, occupational status). Importantly, this association was independent of the presence of parental generalized anxiety disorder symptomatology and the severity of parental bodily symptoms of anxiety. Thus, our results suggest that, as expected, parental everyday-life anxieties likely constitute an independent concept.

In contrast to clinically relevant anxieties, most of these correlations showed slightly smaller effect sizes. This suggests a weaker influence of these everyday-life anxieties on the development of offspring anxieties. Several explanations possibly account for these findings. First, everyday-life anxieties have increased within the past decade and are rather prevalent [18]. Thus, familial exposition to anxiety behaviors might have increased, rendering an influence on offspring anxieties via familial transmission more likely. A number of factors have been discussed to influence familial transmission, including environmental and parenting factors [34, 35]. Familial environment and parenting behavior are among the most important factors [34, 42]; both are influenced by parental anxieties. For example, maternal anxiety is related to increased maternal control, criticism and negativity [42], and families of individuals with anxiety disorders report lower cohesion, emotional expressiveness, and social support [43, 44]. This may lead to a cumulated risk of developing anxiety disorders in offspring. This suggestion is in line with the finding that parental anxiety significantly predicts child anxiety over time [45]. Interestingly, this assumption is also supported by the current data showing that the combination of parental clinical anxieties, parental everyday life anxieties, as well as sociodemographic variables explained about 18% of variance in the occurrence of offspring anxiety symptomatology. Conversely, there are initial indications that the reduction of parental anxiety symptoms through psychotherapy leads to fewer anxiety symptoms in their children [46], supporting the hypothesis that parental (model) behavior contributes significantly to the transmission of fears to the child.

### Limitations and Future Directions

Although the current effect sizes were partly rather small, the large sample size guarantees sufficient statistical power. Thus, also smaller effects can be considered significant. It seems plausible that a number of minor influencing factors add to the transmission of anxiety within families and larger effects might have already been identified in previous studies [18]. Moreover, our data are comparatively new (i.e., about 10 years after the first wave of the BELLA study) and assess a broad range of anxiety symptomatology, which (together with sufficient statistical power) enables the testing of multifinality, equifinality, and concordance among anxiety disorders.

One limitation of the present study is that offspring anxieties were assessed via parental report only. Indeed, multi-informant assessment is the gold standard for assessing clinical impairments in children since cross-informant agreement is mostly only moderate [47, 48]. Each informant contributes unique information to the understanding of child symptomatology, however, informant validity is still a matter of debate [48]. Thus, it cannot be ruled out that the use of exclusive parental report increases the chance that observed childhood anxiety symptoms are amplified by, or a reflection of, parental anxiety about those symptoms in their children. Future work is needed to determine whether the present findings also hold when using a multi-informant approach [48].

Of course, the restriction of the correlative design applies here, too. Nonetheless, the present data provide reference points for the planning of experimental follow-up studies, where the systematic examination of interventions to reduce risk factors or to increase protective factors may substantiate causal statements. For example, future research should address the significance of the current findings with respect to the treatment of childhood anxiety disorders. We found that enhanced parental everyday-life anxieties, along with enhanced parental clinical anxiety level, influence offspring DSM-IV anxiety disorder symptomology. Previous research has shown that familial factors [49], including parental anxiety [50], may worsen treatment outcome for children with anxiety disorders. Conversely, it has been shown that addressing parental anxiety during treatment of childhood anxieties enhances the outcome of childhood treatment [50]. Thus, future studies should address the impact of everyday-life anxieties on treatment outcome for children with anxiety disorders, as well as investigate the benefit of addressing these anxieties during the course of the treatment process. Finally, we did not assess the frequency of life-events.

Thus, we cannot entirely rule out the possibility that everyday-life anxieties may in part reflect realistic concerns and stress due to the impact of stressful social environments or life events, rather than constituting an independent anxiety domain. However, the impact of life-events and other adverse incidences on familial environments largely dependent on the family’s socioeconomic status (SES). For example, people with lower SES are typically more affected by life-events [51], and daily stressors reported by people with lower SES are typically more severe than those reported by people with higher SES [52].

Although we controlled for SES, a factor that directly corresponds to the frequency of adverse incidences in familial environments, we are cannot entirely rule out the possibility that individual’s ratings of everyday-life anxieties, and the relationship with offspring anxiety, may be influenced by realistic concerns and stress within these families’ lives. Future research should incorporate a direct measure of familial stressful environments and life-events to more thoroughly asses their impact on the association of parental everyday-life anxiety and childhood anxiety symptomatology.

## Summary

The current study shows that approximately 50% of children were free of anxiety symptomatology, 40% showed low symptom load and 12% were moderately or severely strained by current anxiety symptomatology within the last 12 months. Female sex, low socioeconomic status and younger age predicted higher symptom load in children. Offspring symptom severity was strongly predicted by adult anxieties with clinical content. For the first time, the current study showed a relationship between adult everyday-life anxieties and offspring clinical symptomatology. Together, demographic variables and parental anxiety markers explained 18% of the variance in offspring symptom severity. Prevalence rates in younger children have only rarely been assessed [53]. Moreover, no study to date assessed the relationship between adult everyday-life anxieties and offspring anxiety symptomatology. Thus, in sum, the present research gives new insights into the distribution of childhood anxiety symptoms and especially into the relationship between parental and offspring anxieties within the general population.

## Footnotes

1 The data on frequency of symptoms of adult DSM-IV disorders can be found elsewhere [18].

2 The question was added after the assessment had already begun.
