# Correction to: Your Fear is My Fear: The Relationship Between Parental and Offspring Anxieties

**DOI:** 10.1007/s10578-021-01207-5

**Published:** 2021-06-25

**Authors:** Dirk Adolph, Jürgen Margraf, Silvia Schneider

**Affiliations:** Mental Health Research and Treatment Center, Ruhr-University Bochum, Massenbergstrasse 9-13, 44847 Bochum, Germany

## Correction to: Child Psychiatry & Human Development 10.1007/s10578-020-01060-y

The article “Your Fear is My Fear: The Relationship Between Parental and Ofspring Anxieties”, written by Dirk Adolph, Jürgen Margraf, Silvia Schneider, was originally published electronically on the publisher’s internet portal (https://link.springer.com/article/10.1007/s10578-020-01031-3) on 18 September 2020 without open access. With the author(s)’ decision to opt for Open Choice, the copyright of the article changed on 8 June 2021 to © The Author(s) 2021 and the article is forthwith distributed under the terms of the Creative Commons Attribution 4.0 International License (http://creativecommons.org/licenses/by/4.0/), which permits use, duplication, adaptation, distribution and reproduction in any medium or format, as long as you give appropriate credit to the original author(s) and the source, provide a link to the Creative Commons license and indicate if changes were made.

The Open Access funding enabled and organized by Projekt DEAL.

The original article has been corrected.

